# Defining the minimum inhibitory concentration of 22 rifamycins in iron limited, physiologic medium against Acinetobacter baumannii, Escherichia coli, and Klebsiella pneumoniae clinical isolates

**DOI:** 10.1371/journal.pone.0287102

**Published:** 2023-06-13

**Authors:** Peggy Lu, Francesco Alletto, Bosul Lee, Elizabeth Burk, Jasmine Martinez, Fabio Prati, Emilia Caselli, Brad Spellberg, Brian Luna

**Affiliations:** 1 Department of Molecular Microbiology and Immunology, Keck School of Medicine at USC, Los Angeles, California, United States of America; 2 Department of Life Science, University of Modena and Reggio Emilia, Modena, Italy; 3 Los Angeles General Medical Center, Los Angeles, California, United States of America; Cornell University, UNITED STATES

## Abstract

Recently, we reported rifabutin hyper-activity against *Acinetobacter baumannii*. We sought to characterize if any additional rifamycins (n = 22) would also display hyper-activity when tested in iron-limited media against *A*. *baumannii*, *K*. *pneumoniae*, and *E*. *coli*. MICs were determined against representative clinical isolates using the iron-limited media RPMI-1640. Only rifabutin was hyperactive against *A*. *baumannii*.

## Introduction

We previously conducted a modified compound screen assay against *Acinetobacter baumannii* using RPMI-1640 broth, a medium that better models the *in vivo* physiologic blood environment as compared to the rich mediumMueller Hinton II broth, cation-adjusted (MHII)). We found that rifabutin (RBT) possesses previously unrecognized hyper-activity against *A*. *baumannii*, but only in physiological media, and not in rich media [1–3]. RBT is able to rapidly enter the cell through the FhuE protein, an iron transport protein that is downregulated when the bacteria are replete with iron [1,4]. We have previously tested rifabutin, rifampin, rifaximin, rifapentine, FCE-22250, rifalazil, and rifamycin sv against *A*. *baumannii* and only rifabutin was able to traffic through the FhuE protein in iron limited conditions [1]. Here, we sought to determine if any additional, commercially available rifamycins would have increased activity in iron limited conditions when tested against representative isolates of *A*. *baumannii*, *Klebsiella pneumoniae*, and *Escherichia coli*.

### Rifamycin MIC distributions

**Fig 1:**
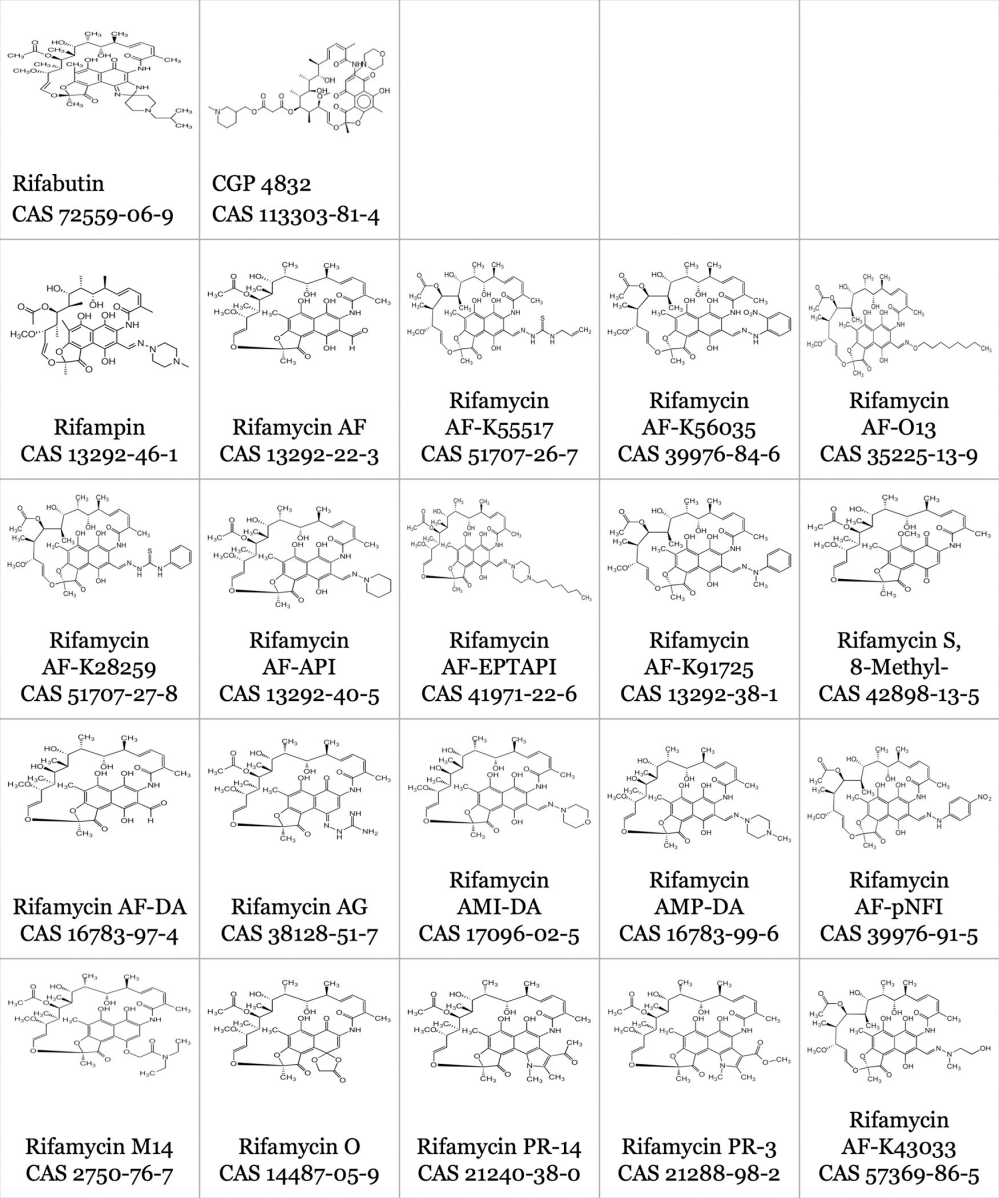
Description of compounds tested.

We determined rifamycin MICs (n = 185) against a larger panel of 21 rifamycin compounds against *A*. *baumannii* (n = 5), *K*. *pneumoniae* (n = 3), and *E*. *coli* (n = 1) clinical isolates using the broth microdilution method per the Clinical & Laboratory Standards Institute (CLSI), but modified to use the iron-limited medium RPMI-1640 as previously described [1–3,5,6]. A summary of the compounds tested is listed in **Table 1.** Consistent with our previous data, we did observe hyper susceptible RBT phenotypes (MIC < 0.05 mg/L) for the *A*. *baumannii* HUMC1 and ATCC17978 isolates [1,2]. However, no isolate for any species tested was hypersusceptible to any other compound tested **(Table 1, Fig 1)**.

**Table 1:** MICs for rifamycin compounds determined in RPMI-1640.

Drug	AB HUMC1	AB ATCC 17978	Ab 5075	Ab 6049	AB LAC-4	EC JJ1886	KP KPC- KP1	KP KP3	KP KP4
Rifabutin	**0.0625**	**0.0625**	**0.0625**	**0.0625**	1	8	16	16	16
Rifampin	8	8	4	2	1	32	>32	32	32
Rifamycin AF	>32	32	ND	16	2	16	>32	32	32
Rifamycin AF-K4033	8	4	ND	8	8	8	>32	32	32
Rifamycin AF-K55517	16	8	16	32	8	32	>32	>32	>32
Rifamycin AF-K56035	32	32	16	32	32	32	>32	>32	>32
Rifamycin AF-K28259	4	16	8	32	4	32	>32	>32	>32
Rifamycin AF-API	16	16	8	8	1	8	>32	32	>32
Rifamycin AF-EPTAPI	>32	>32	32	ND	>32	16	>32	>32	>32
Rifamycin AF-K91725	16	8	16	4	8	16	>32	32	>32
Rifamycin AF-DA	>32	>32	>32	32	>32	>32	>32	>32	>32
Rifamycin AF-O13	>32	>32	>32	32	>32	>32	>32	>32	>32
Rifamycin AF-pNFI	32	>32	>32	16	>32	32	>32	>32	>32
Rifamycin AG	2	1	2	2	1	2	16	8	16
Rifamycin AMI-DA	>32	>32	4	32	>32	32	>32	>32	32
Rifamycin AMP-DA	>32	32	32	32	32	16	32	>32	>32
Rifamycin M14	>32	32	>32	32	>32	16	>32	>32	>32
Rifamycin O	16	16	8	8	32	16	>32	>32	>32
Rifamycin PR-14	>32	>32	16	32	>32	16	32	>32	32
Rifamycin PR-3	16	16	8	8	16	8	32	4	16
Rifamycin S, 8-methyl-	32	16	>32	32	32	16	>32	>32	>32

### CGP-4832

Previous studies have shown that the rifamycin CGP-4832 is able to traffic through the *E*. *coli* iron transport protein FhuA [7–9]. CGP-4832 MICs against *E*. *coli* MG1655 were 8 mg/L and 0.25 mg/L in MHII and RPMI-1640 respectively. This 32-fold shift in MIC is consistent with the described mechanism of action for CGP-4832 against *E*. *coli*. As this mechanism is conceptually similar to the mechanism described for RBT entry, we tested if *A*. *baumannii* is hyper susceptible to CGP-4832. However, no benefit was observed for *A*. *baumannii*. CGP-4832 MICs against *A*. *baumannii* HUMC1 and LAC-4 were 2 mg/L and 4 mg/L respectively in MHII; and 8 mg/L and 2 mg/L respectively in RPMI-1640.

**Fig 2:**
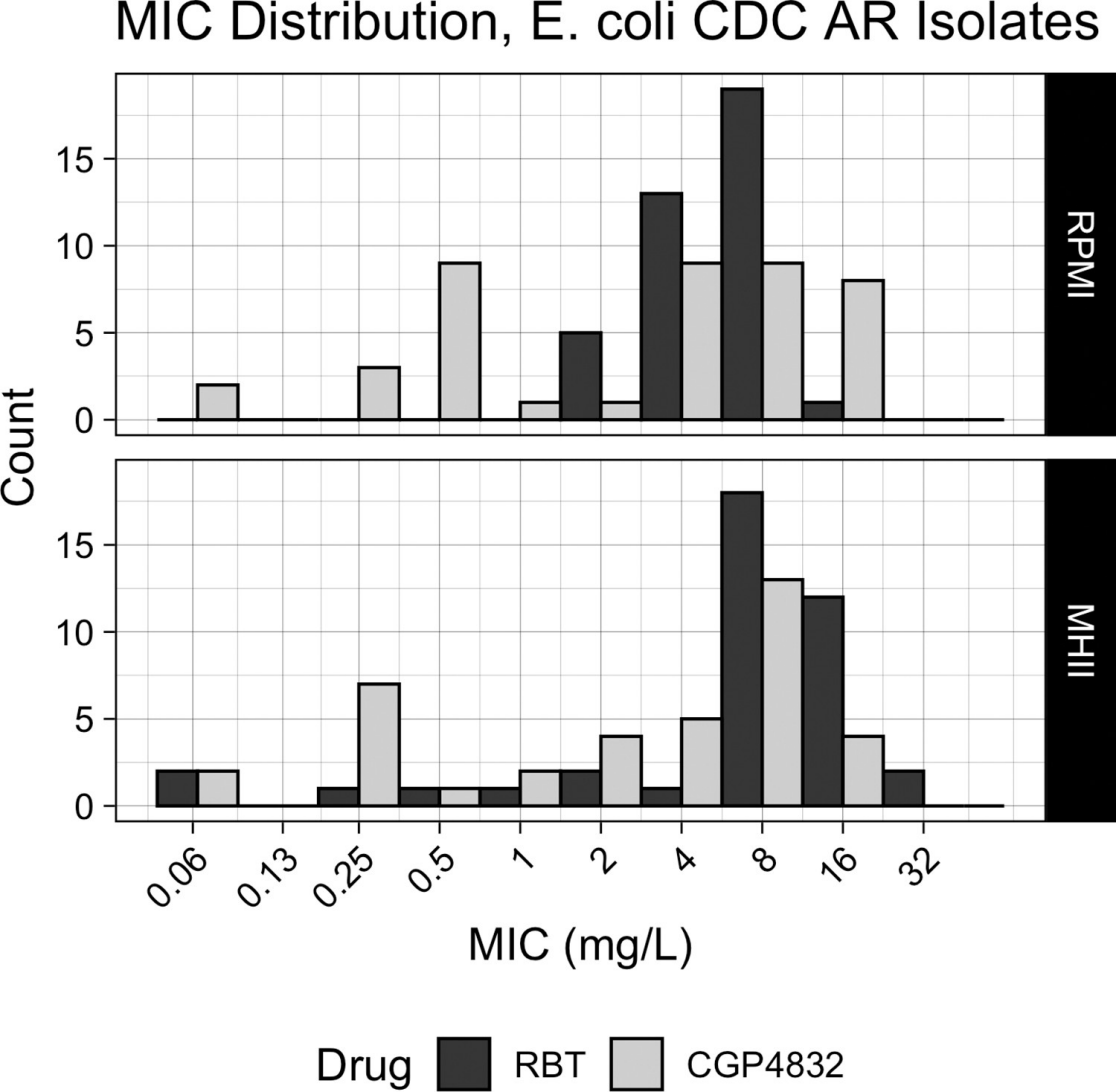
Distribution of CGP-4832 MICs determined in MHII or RPMI-1640 media against 45 *E*. *coli* clinical isolates.

It was previously estimated that about 33% of clinical isolates were hypersusceptible to CGP-4832 [7]. However, this number was determined using only MHII for susceptibility testing because it was not yet known that transport of CGP-4832 through the FhuA protein depended on an iron-limited environment. CGP-4832 MICs were determined against a panel of 45 *E*. *coli* clinical isolates, obtained from the CDC and FDA AR Isolate Bank, in MHII or RPMI-1640 to test if a greater proportion of clinical isolates would be hypersusceptible to CGP-4832 if MICs were determined in iron-limited media. There was a significant difference in CGP-4832 and RBT MICs determined in MHII (p = 0.001, Mann-Whitney), but no significant difference when MICs were determined in RPMI-1640. Additionally, there was not a statistically significant difference between the distribution of GCP-4832 MICs determined in the iron-rich MHII as compared to the iron-limited RPMI-1640 media (**Fig 2, S1 Table).** This result was somewhat surprising because sideromycin antibiotics such as albomycin, also dependent on transport by FhuA, displayed increased activity in iron-limited media [10].

## Conclusions

The use of culture media that mimics the *in vivo* physiologic environment during infection is a valuable tool for characterizing antibiotic activity *in vitro*. Rifabutin and CGP-4832 are the only rifamycin compounds that are able to traffic through the FhuE protein of *A*. *baumanii* or the FhuA protein of *E*. *coli* respectively. We have previously shown that rifabutin is not able to enter *E*. *coli* through FhuA of *E. coli [2]*. The library of compounds tested represents broad chemical diversity and will further guide the development of additional derivatives capable of being transported more broadly by both FhuA and FhuE proteins of diverse bacterial species.

## Methods

### MIC assay

Unless otherwise indicated, the standard broth microdilution method following CLSI methodology was used to determine MICs [5]. The media used for the minimum inhibitory concentration (MIC) assays performed in this study were MHII or RPMI-1640 as previously described [1–3,6,11,12].

Briefly, 100 μL of media was added to the wells in columns 2–10. Column 11 served as a positive growth control and contained only bacteria and media. Column 12 served as the sterility control and contained only culture media without bacteria. Next, 200 μl of a 2X antibiotic working solution was added to the wells in column 1. Two-fold serial dilutions of the antibiotic were performed through column 10. Next, 100 μl of a 1×10⁶ CFU/mL working solution of bacteria were added to each of the wells in columns 1–11. The inoculum concentration was confirmed by plating serial dilutions on TSA plates. MIC plates were incubated at 35±2˚C and results were recorded at 24 hours.

## Supporting information

S1 Table MIC summary. Distribution of CGP-4832 MICs determined in MHII or RPMI-1640 media against 45 *E*. *coli* clinical isolates.
(CSV)
